# Determinants of long acting contraceptive utilization among HIV positive reproductive age women attending care at art clinics of public health facilities in Arba Minch town, Southern Ethiopia, 2019: a case control study

**DOI:** 10.1186/s12981-020-00288-x

**Published:** 2020-06-15

**Authors:** Betselot Yirsaw, Feleke Gebremeskel, Gebrekiros Gebremichael, Tewoderos Shitemaw

**Affiliations:** 1 College of Health Science, Addis Ababa University, Addis Ababa, Ethiopia; 2 Department of Public Health, Arba Minch University, Arba Minch, Ethiopia; 3 Department of Anesthesia, Menelik II College of Medical & Health Science, Kotebe Metropolitan University, Addis Ababa, Ethiopia

**Keywords:** Long acting contraceptive methods, HIV positive reproductive age women, Ethiopia

## Abstract

**Background:**

Long acting and permanent contraceptive methods by far are the most effective, very safe and convenient methods than short acting contraceptive methods. But in less developed countries, use of long acting reversible contraceptive or permanent methods (LARCs/PMs) is very low. Therefore the aim of this study was to identify determinants of long acting contraceptive method utilization among HIV positive reproductive age women.

**Methods:**

An institutional based case control study was conducted among random sample of 354 HIV positive reproductive age women (total of 97.8% response rate) at Anti-Retroviral Therapy clinics from February 20 to March 20, 2019. Case to control ratio was 1:2. A structured questionnaire and information recorded from ART card review were used to collect the data. Each variable was entered in Bivariate analysis with dependent variables and those variables with P-value of ≤ 0.25 were included in the Multivariate analysis. Significance was determined at the level of P-value < 0.05 with 95% CI of AOR.

**Results:**

A total of 354 (33.3% cases and 66.7% controls) HIV positive reproductive age women were interviewed with response rate of 97.8%. The study revealed being in age group of 39 and above [AOR = 0.17, 95% CI (0.06, 0.48)], being divorced/separated and widowed [AOR = 0.05, 95% CI (0.003, 0.61)], having supportive opinion and strongly supportive opinion regarding family planning service availability in ART clinic [AOR = 5.01, 95% CI (1.79, 14.07)], [AOR = 7.81, 95% CI (2.54, 24.01)] and having no future fertility intention [AOR = 7.03, 95% CI (2.73, 18.06)] were statistically significant determinants for long acting contraceptive method utilization.

**Conclusion:**

Woman in age group of 39 and above, having no future fertility intention and being divorced/separated and widowed was found to be determinants of long acting contraceptive method utilization among HIV positive reproductive age women. In addition our study support the WHO Strategic Considerations for Strengthening the Linkages between Family Planning and HIV/AIDS Policies, Programs, and Services.

## Introduction

All individuals and couples have a basic human right to decide freely and responsibly the number, spacing and timing of their children [1, 2]. Fulfilling this right is an important intervention for improving maternal and child health, preventing HIV infections, and improving the overall well-being of entire families [3]. Long acting and permanent contraceptive methods (LAPMs) by far is the most effective (99% or greater), very safe and convenient methods than short acting methods. LAPMs provide 3 to 12 years and life time uninterrupted protection against unintended pregnancy [4, 5]. HIV/AIDS is the most important public health challenge globally, especially the largest burden in Sub-Saharan Africa (SSA) [6]. Dual protective which includes use of a reliable hormonal contraceptive method like LAPMs and a barrier method like using the male or female condom is encouraged to prevent further transmission of HIV [4]. According to the United Nations report, Ethiopia is stated as one of 22 priority countries for eliminating mother-to-child transmission of HIV [7].

Fertility is not affected by HIV infection, however behavioural change, existing subfertility, low body mass index, AIDS, and inter current illness may lower conception rates [8]. Women with HIV infection, like other women, may wish to plan pregnancy, limit their family, or avoid pregnancy [9]. In the least developed countries, use of long acting reversible contraceptive or permanent methods (LARCs/PMs) accounts for less than one-fifth (19%) of the contraceptive method mix [10]. According to different studies done in Ethiopia, LARCs/PMs utilization has been seen very low among HIV positive reproductive age women [11–14]. Hence the aim of this study was to identify the determinants of long acting contraceptive methods utilization among HIV positive reproductive age women attending care in ART clinics in all public health facilities of Arba Minch Town.

## Method and materials

### Study design and setting

An institutional based case control study was conducted in three public health facilities of Arba Minch town, Gamo zone, Southern Ethiopia from February 20 to March 20, 2019. Arba Minch town is the capital city of Gamo zone with 3 public health care facilities (one general hospital and two health centers) and 33 private clinics (18 level one clinics and 15 medium clinics). All 3 public health care facilities are giving ART clinic services for seropositive peoples in the town and surrounding areas.

### Population

All HIV positive reproductive age group women (15–49 years) who had ART follow up in all public health facilities of Arba Minch town were source population and those women who fulfilled the inclusion criteria during the data collection period were study population.

All HIV positive reproductive age women who were using long acting contraceptive methods were considered as cases while all HIV positive reproductive age women who were not using contraceptive methods (non-user) were considered as controls.

All HIV positive reproductive age women who use short acting contraceptive methods and women who were severely ill during data collection period were excluded from the study.

### Sample size determination and Sampling technique

The sample size was determined by using the double population proportion approach using Epi Info version 7.02 statistical software package with the assumption of 95% confidence level (Zα/2 = 1.96), 80% power. Case to control ratio of 1:2 and the sample size was calculated by taking different factors from two different studies done in Bahir Dar North West Ethiopia [11, 13].

The larger sample size which was 344 taken from previous study done in Bahir Dar City, Ethiopia was taken for this study [11]. After adding 5% for non-response rate, the total sample size was 362 (121 cases and 241 controls).

Cases and controls were selected from each health facilities that are giving ART clinic services for Arba Minch town and surrounding areas. The sample was proportionally allocated depending on their 3 month performance before data collection using the patient registry log book at ART clinics. Using systematic random sampling techniques the required sample size were drawn as follows. From Arba Minch General hospital 272 (91 cases and 181 Controls), from Sikela health center 86 samples (29 cases and 57 controls) and from Secha health center 3 samples (1 case and 2 controls) were included in the study.

### Operational definitions and definition of terms

- Long acting contraceptive methods: Modern contraceptive methods that prevent unintended pregnancy for more than 1 year which include Long Acting Reversible contraceptive Methods (LARMs) such as Intra Uterine Devices (IUDs) and sub dermal Implants and permanent contraceptive methods (Tubal ligation) [11].
- Myths heard: When women had ever heard any rumor or misconceptions about LAPMs [13].
- Past experience for LAPMs: It is when a women had ever used LAPMs before current used method [13].
- Current use of contraceptive method: Referred to respondents who responded positively for use of at least one type of contraceptive methods at time of the survey to delay or avoid pregnancy [14].

### Data collection tool, method and procedure

Data was collected using structured questionnaire to interview all eligible women and data extraction tool which was adapted from previous study and some adjustment were made for this study [15]. The questionnaire mainly addressed socio demographic variables of mothers (age, women’s educational status, husband educational status, marital status, wealth index and place of residence), Reproductive health variables (parity, gravidity, number of live children, discussion with husband, discussion with health care providers, family planning counseling, pregnancy intention, experience of contraceptive) and Risky sexual behaviour variables (sexual intercourse in the past 6 months, using condom, having multiple sexual partner). Data extraction tool was used for Medical history variables (clinical WHO staging, disclosure of HIV status, CD4 count, time of HIV diagnosed and HAART user). Five nurses and one public health officer participated as data collectors and supervisor, after training and orientation had given for 2 days. The principal investigator checked the completeness of the data each day.

### Data quality control

Data collectors and the supervisor were trained for 2 days on data collection tool and the procedure by the principal investigator. Pre-test was done in Chencha hospital 1 week before the actual data collection, and few amendment were made after the pre-test. The required data were collected after obtaining ethical clearance from Arba Minch University Institutional Review Board (AMU/IRB). In addition permission from the three health institutions and consent from the patient was taken. The entire questionnaire was checked and reviewed for completeness and consistency every day by principal investigator before data entry.

### Data analysis

After the data collection, data was coded and then entered, cleaned and edited by Epi Info version 7 and exported to Statistical package for Social Sciences (SPSS) software version 23. Wealth index of the participants was analyzed by principal component analysis method. On binary logistic regression analysis, a variable with P-value of ≤ 0.25 was used as a candidate for multiple logistic regression analysis. Multicollinearity was checked by using standard error greater than two. Multi variable logistic regression with backward method was done to find determinant factors. Odds ratio, 95% confidence interval and P-value < 0.05 were used to determine the significance and strength of association with dependant variable.

### Ethical considerations

Ethical approval and clearance were obtained from an ethical review committee of Arba Minch University, College of Medicine and Health sciences. Further permission was obtained from all public health facilities. Confidentiality was maintained by making the data collectors aware not to record any identification information found.

## Results

### Socio demographic and economic characteristics of the participants

The total number of women visited the art clinic during the study period was more than 800. Of which, 362 HIV positive reproductive age women were participated in the study. 354 HIV positive reproductive age women were included in the final analysis with a response rate of 97.8% in the study and 8 women excluded due to non-response or incomplete data. Among those 118 (33.3%) cases and 236 (66.7%) controls were responded to the interview respectively. 83 (70.3%) cases and 139 (58.9%) controls were in the age category of 29-38 years. Orthodox was the predominant religion for cases 67 (56.8%) and controls 136 (57.6%). Of the total 91 (77.1%) cases and 104 (44.1%) controls were married. Concerning their educational status 16 (13.6%) cases and 28 (11.9%) controls were attended above secondary level and regarding their husbands educational status 19 (17.6%) cases and 23 (18.7%) controls attend above secondary level. Regarding the Economic status of the respondents, around one fifth of cases and controls were in the lowest wealth quantile (Table 1).

**Table 1:** Socio demographic and economic characteristics among HIV positive reproductive age women attending at ART clinics

Variables	Category	Cases (n = 118)		Controls (n = 236)	
		Number	Percent (%)	Number	Percent (%)
Age in years	19–28	30	25.4	49	20.8
	29–38	83	70.3	139	58.9
	39 and above	5	4.2	48	20.3
Religion status	Orthodox	67	56.8	136	57.6
	Muslim	8	6.8	17	7.2
	Protestant	43	36.4	83	35.2
Place of residence	Rural	14	11.9	32	13.6
	Urban	104	88.1	204	86.4
Marital status	Single	3	2.5	19	8.1
	In relationship/union	17	14.4	19	8.1
	Divorced/separated and widowed	7	5.9	94	39.8
	Married	91	77.1	104	44.1
Educational level of women	No formal education	36	30.5	79	33.5
	Primary	38	32.2	77	32.6
	Secondary	28	23.7	52	22.0
	Above secondary	16	13.6	28	11.9
Occupation of women	House wife	37	31.4	67	28.4
	Daily laborer	21	17.8	49	20.8
	Own business	15	12.7	51	21.6
	Government/private employee	36	30.5	53	22.5
	Others^a^	9	7.6	16	6.8
Wealth index	Lowest	24	20.3	48	20.3
	Second	21	17.8	44	18.6
	Medium	30	25.4	46	19.5
	Fourth	22	18.6	48	20.3
	Highest	21	17.8	50	21.2

Women occupation—others^a^: commercial sex workers, students and farmers

### Reproductive health related characteristics among HIV positive reproductive age women

Among the total cases, the majority of the respondents were using implanon 98 (83.1%) followed by intrauterine device 14 (11.9%) and Tuba-ligation 6 (5.1%). Of the total cases 71 (61.2%) women were utilizing dual contraceptive methods. Two third of the cases (67.5%) were using the current long acting contraceptive for 1 to 3 years. Among the respondents, 74 (62.7%) of cases and 115 (48.7%) of controls get pregnant after becoming HIV positive and for both groups more than 90% women become pregnant only 1–2 times (Table 2).

**Table 2:** Reproductive health related characteristics among HIV positive reproductive age women attending care at ART clinics

Variables	Category	Cases (n = 118)		Controls (n = 236)	
		Number	Percent (%)	Number	Percent (%)
Have children	Yes	108	91.5	191	80.9
	No	10	8.5	45	19.1
Number of children (n = 299)	1–2	49	45.4	104	54.5
	3–4	44	40.7	69	36.1
	5 and above	15	13.9	18	9.4
Pregnancy after becoming HIV positive	Yes	74	62.7	115	48.7
	No	44	37.3	121	51.3
Number of pregnancy after becoming HIV positive (n = 189)	1–2	68	91.9	108	93.9
	3–4	6	8.1	7	6.1
Contraceptive use before HIV diagnosed	Yes	65	59.9	111	47
	No	53	44.1	125	53
Myth and misconception about LACMs	No	118	100	30	12.7
	Yes	0	0	206	87.3
Opinion regarding family planning service availability in ART clinic	Neutral	27	22.9	134	56.8
	Support	48	40.7	66	28.0
	Strongly support	43	36.4	36	15.3
Counseled about contraceptives by ART providers	No	0	0	25	10.6
	Yes	118	100	211	89.4

**Fig. 1:**
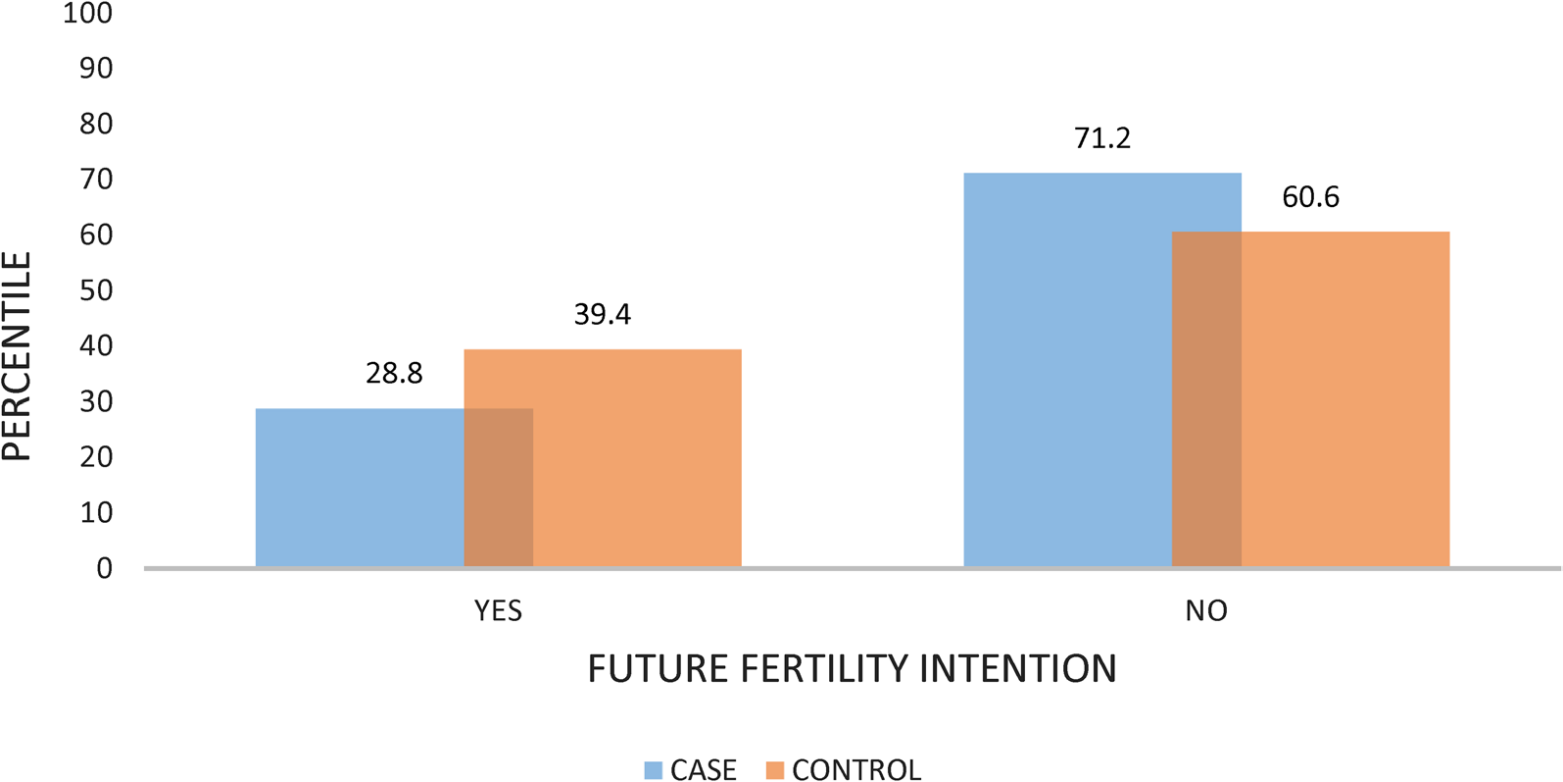
Future fertility intention among HIV positive reproductive age group attending care at ART clinics

Of the total participants 84 (71.2%) cases and 143 (60.6%) controls report that they do not have future fertility intention (Fig. 1).

### Medical history related and risky sexual behaviour characteristics among HIV positive reproductive age women

This study indicate that nearly two third of the cases (68.6%) and 134 (56.8%) controls diagnosed HIV with in the last 5 years. Regarding the ART initiation, 76 (64.4%) cases and 128 (54.2%) controls start ART drug with in the last 5 years. Nearly 72 (65%) cases and 141 (66.5%) controls have CD4 count greater than 500 mm/cells and 97.5% of cases and 96.2% of controls were in WHO stage one.

Out of the total respondents 107 (90.7%) cases and 198 (83.9%) controls had information about mother to child transmission of HIV. More than half of the respondents (60.7% cases and 60.1% controls) report health care providers in ART clinic were the main source of information followed by mass media (13.1% cases and 9.1% controls). Only 30 (28.0%) of cases and 54 (27.3%) of controls have information that HIV can transmit during pregnancy, delivery and breast feeding (Table 3).

**Table 3:** Medical history and risky sexual behavior characteristics among HIV positive reproductive age women at ART clinics

Variables	Category	Cases (n = 118)		Controls (n = 236)	
		Number	Percent (%)	Number	Percent (%)
ART drug started time in years	≤ 5	76	64.4	128	54.2
	> 5	42	35.6	108	45.8
CD4 count in cells/mm^3^	< 500	39	35.1	71	33.5
	≥ 500	72	64.9	141	66.5
WHO staging	Stage 1	115	97.5	227	96.2
	Stage 2	2	1.7	4	1.7
	Stage 3	1	0.8	5	2.1
HIV transmit from mother to child	No	11	9.3	38	16.1
	Yes	107	90.7	198	83.9
Use condom during sexual intercourse in the previous 6 months	Yes	71	61.2	78	36.8
	No	45	38.8	134	63.2
Have multiple sexual partner	Yes	8	6.8	11	4.7
	No	110	93.2	225	95.3

### Determinant factors of long acting contraceptive utilization among HIV positive reproductive age women

In the final model of this study, the determinant factors which were statistically significant with long acting contraceptive utilization among HIV positive reproductive age women attending care at ART clinics in all public health facilities of Arba Minch town were age, Marital status, future fertility intention and opinion about family planning service availability in ART clinic.

Being in 39 and above age group were found to be associated with long acting contraceptive method utilization with the odds of 90% less likely to use long acting contraceptive than those who were in age group of 19–28 years old women [AOR = 0.10, 95% CI (0.02, 0.54)].

Marital status of being divorced/separated and widowed as compared to married women were associated with long acting contraceptive utilization with the odds of 95% [AOR = 0.05, 95% CI (0.003, 0.61)] (Table 4).

**Table 4:** Determinants of long acting contraceptive utilization among HIV positive reproductive age women attending care at ART clinics

Variables	Category	Cases	Controls	COR (95% CI)	AOR (95% CI)
Age	19–28	30 (25.4%)	49 (20.8%)	1	1
	29–38	83 (70.3%)	139 (58.9%)	0.98 (0.57, 1.66)	0.91(0.28, 2.87)
	39 and above	5 (4.2%)	48 (20.3%)	0.17 (0.06, 0.48)	0.10 (0.02,0.54)**
Marital status	Single	3 (2.5%)	19 (8.1%)	1	1
	In relationship/union	17 (14.4%)	19 (8.1%)	5.67 (1.42, 22.6%)	4.36 (0.58, 33.09)
	Divorced/separated and widowed	7 (5.9%)	94 (39.8%)	0.47 (0.11, 1.99)	0.05 (0.003, 0.61)**
	Married	91 (77.1%)	104 (44.0%)	5.54 (1.59,19.34)	3.34 (0.63, 17.74)
Opinion on availability of FP service in ART clinic	Neutral	27 (22.9%)	134 (56.0%)	1	1
	Support	48 (40.7%)	66 (28.0%)	3.61 (2.07,6.29)	5.01 (1.79, 14.07)**
	Strongly support	43 (36.4%)	36 (15.3%)	5.92 (3.24,10.86)	7.81 (2.54, 24.01)**
Future fertility intention	No	84 (71.2%)	143 (60.6%)	1.61 (0.99, 2.59)	7.03 (2.73, 18.06)**
	Yes	34 (28.8%)	93 (39.4%)	1	1

** P < 0.05

## Discussion

This study finding revealed that women aged 39 years old and above, divorced/separated and widowed women, women who support and strongly support availability of family planning method in ART clinic and women who have no future fertility intention were associated with long acting contraceptive utilization.

Women aged 39 years old and above were 90% less likely to use long acting contraceptive methods. This finding is consistent with a study conducted at University of Gonder hospital and Mizan Tepi teaching and referral hospital which indicated that reproductive age women who were 35 years old and above were 83% and 70% less likely to use modern contraceptive methods [14, 16]. This could be explained as majority of women (60.4%) who were 39 and above years of age in this study were divorced/separated and widowed. In addition women 39 and above years of age did not use long acting contraceptive methods due to expectation of the natural cessation of menses and due to fear of side effects and misconceptions. Commonly reported Misconceptions and fear of side effects mentioned in another study done in Southern Ethiopia included headache, disturbance of menstruations, weight gain, nausea, loss of appetite, infertility and other medical problems like hypertension, anemia, cancer, kidney stone and other disease after long use [17].

According to this study women who were divorced/separated and widowed were associated with long acting contraceptive utilization compared to single women with the odds of 95%. This result is supported by a study conducted in Mizan Tepi, Southern Ethiopia indicate women who were divorced, separated or widowed were 79% less likely to use modern contraceptive than women who were married [14]. The result is also consistent with studies conducted at university of Gonder hospital and Addis Ababa, [16, 18]. This may be due to above half of them were in age group of 39 and above years of age and they may not need long acting contraceptive methods to prevent unwanted pregnancy. In addition majority of women in this group had neutral opinion on the availability of family planning service in ART clinic.

As this study indicated women who had no future fertility intention were seven times more likely to use long acting contraceptive than those women who had fertility intention. This finding is supported by study conducted in Bahir Dar, Ethiopia, on demand of long acting contraceptive among married HIV positive women, it showed women who had no fertility intention were 7.7 times more likely to had intension to use long acting contraceptive [11] similarly studies conducted in Arba Minch Zuriya District, in Bahir Dar town and indicate the same [19, 20]. This may be due to women who had no fertility intention prefer long acting contraceptive methods which is the more effective contraceptive methods.

This study also found women who have supportive and strongly supportive opinion on the availability of family planning service in ART clinic were five times and eight times more likely to use long acting contraceptive than those who have neutral opinion. A longitudinal cohort study done in Malawi [21] on integrating family planning service into HIV care also demonstrate the rapid increase in contraceptive use during implementation of enhanced Family Planning-Electronic Medical Record (FP EMR) module, possibly due to increased counselling and FP-related communications with the women [2].

The above result strongly support the WHO’s Strategic Considerations for Strengthening the Linkages between Family Planning and HIV/AIDS Policies, Programs, and Services. The strategy indicate integration of family planning at HIV service delivery points providing a valuable opportunity to comprehensively address the risk of HIV infection, prevent unintended pregnancy and promote healthy birth spacing and birth outcomes [2].

### Limitation of study

The limitation of the study was that the study relied on participants’ self-reported data, which was prone to social desirability bias. However, close monitoring by supervisor and PI was made to minimize such biases and clarification of potential ambiguities and misunderstandings, maintaining privacy of participants during interview carried out by interviewers. In addition, data lack adequate published litratures on same study design to compare detrminant variables more.

## Conclusion

Family planning (FP) is one aspect of RH where linkages with HIV programs are especially important. Integrating FP services into HIV prevention, treatment, and care services provides an opportunity to increase access to contraception among clients of HIV services who do not want to become pregnant, or to ensure a safe and healthy pregnancy and birth for those who wish to have a child [2].

This study identified having supportive and strongly supportive opinion on availability of family planning service in ART clinic and had no future fertility intention were positively associated with long acting contraceptive utilization while being in age group of 39 and above years old and being divorced/separated and widowed were negatively associated with long acting contraceptive utilization among HIV positive reproductive age women.

## Data Availability

The data used in this study was collected by trained data collectors and the lead author are willing to share the data upon request from peer researchers.

## Abbreviations

AOR: Adjusted odds ratio
AIDS: Acquired immunodeficiency syndrome
ART: Ant retro viral therapy
FP: Family planning
HAART: Highly active antiretroviral therapy
HIV: Human immune deficiency virus
IUDs: Intra uterine devices
LAPMs: Long acting and permanent contraceptive methods
LARMs: Long acting reversible contraceptive methods
PM: Permanent methods
PMTCT: Prevention of mother to child transmission
RH: Reproductive health
SSA: Sub-Saharan Africa
